# Impact of the Covid-19 pandemic on people with a pre-existing psychiatric disorder

**DOI:** 10.1192/j.eurpsy.2022.1383

**Published:** 2022-09-01

**Authors:** S. Khouadja, M. Asgharzadeh, S. Younes, L. Zarrouk

**Affiliations:** University Hospital of Mahdia, Tunisia, Psychiatry, Mahdia, Tunisia

**Keywords:** psychiatry, Covid-19, schizophrénia

## Abstract

**Introduction:**

The Covid-19 pandemic has had a deleterious impact on populations across the world. Yet it remains unclear how this pandemic is affecting the psychological health of people with a previous history of mental illness.

**Objectives:**

This study aims to investigate the impact of Covid-19 on patients with established mental disorders.

**Methods:**

The PubMed and science direct databases were systematically searched using the keywords combination “Covid-19” and “psychiatric disorders”, “the pandemic” and “mental disorders “, from inception up to November 2021. We adopted a broad inclusion criterion for the study requiring patients to have a pre-existing mental disorder, excluding narrative reviews and preclinical studies. In addition, a search of google scholar was conducted to identify any additional relevant publications.

**Results:**

We have found 26 studies but only 19 met our inclusion criteria. Included studies were published between 2020 and 2021. 2 major results were identified. Symptoms deterioration was reported in individuals with severe mental disorders and those with schizophrenia in particular, such as depressive or anxiety symptoms, substance use and suicidal ideation, due to the psychological stress and physical distancing measures associated with the Covid-19 outbreak. The symptomatic treatments used in Covid-19 had frequent interactions with the most used antipsychotic drugs leading to a substantial increase in relapse rates in people with mental disorders.

**Conclusions:**

The Covid-19 pandemic has a serious impact on individuals with pre-existing mental illness reinforcing symptom severity and psychological stress. Additional studies are needed to strengthen current findings with pre-pandemic records.

**Disclosure:**

No significant relationships.

